# Supplementary material for: Are carbon emissions trading and green financial instruments synergistic? -Comprehensive quantitative research based on content analysis
Source: PLoS One. 2024 Mar 7;19(3):e0298601. doi: 10.1371/journal.pone.0298601 (PMC10919601; doi:10.1371/journal.pone.0298601)
Supplement: S1 Data — (ZIP) [file pone.0298601.s001.zip › Data/地级市Z_F工作报告.zip!/─┌├╔╣┼/║⌠┬╫▒┤╢√╩╨/└┤╫╘╣1⁄2╓┌║┼í╛┬φ┐╦╩2╛▌═°í┐_2006.docx]

各位代表：

现在，我代表呼伦贝尔市人民政府向大会作报告，请连同《呼伦贝尔市国民经济和社会发展第十一个五年规划纲要（草案）》一并审议，并请呼伦贝尔市政协委员和列席会议的同志们提出意见。

一、“十五”时期和2005年经济社会发展回顾

“十五”时期，是呼伦贝尔市经济摆脱困境、夯实基础、谋求突破和各项社会事业快速发展的五年。五年来，全市上下以邓小平理论和“三个代表”重要思想为指导，认真贯彻落实科学发展观，解放思想，抢抓机遇，开拓创新，真抓实干，成功地战胜了洪涝、干旱、“非典”、“禽流感”等多种重大自然灾害，完成了撤盟设市的重大体制转变，巩固了民族团结、边疆稳定、社会和谐的良好局面，圆满完成了“十五”计划确定的各项奋斗目标。

（一）综合经济实力明显增强

五年来，全市上下始终坚持以经济建设为中心，以结构调整为主线，以建设“三大基地”、“七大产业集群”为目标，抢抓综合机遇，狠抓项目建设，综合经济实力明显增强。全市地区生产总值中一、二、三次产业比例由2000年的33．6∶20．2∶46．2调整为2005年的28．0∶28．6∶43．4。全市地区生产总值由157亿元增加到319．2亿元，比2000年增长1倍，年均增长15．2％；人均生产总值达到11717元（折合1446美元），比2000年增长1倍，年均增长15．1％；财政收入达到35．72亿元，比2000年增长1．3倍，年均增长17．8％，财政总支出达到61．12亿元，比2000年增长2．7倍，年均增长22．34％；固定资产投资累计完成555．9亿元，超过前九个五年计划的投资总和，年均增长33．8％。具有我市特色和比较优势的能源重化工业、畜牧业、旅游业拉动三次产业快速增长的产业发展格局初步形成。

（二）改革和开放进一步深入

在改革上，进一步加大了企业改制重组、优化整合力度，推进了煤炭、农垦、铁路、地方林业等国有大企业剥离办社会工作；农村牧区各项改革稳步实施，在全市范围内全部免除了农牧业税，农村金融体制改革、粮食流通体制改革迈出了新步伐；行政管理体制改革成效显著，行政审批事项由615项减少到184项，精简比例达到70％，进驻行政审批服务中心的办理事项达到100％。在开放上，狠抓了招商引资工作，神华集团、鲁能集团、中集集团、瑞士雀巢、香港嘉里等一批国内外知名的大企业、大集团入驻我市，企业的市场竞争力和发展活力明显增强。五年累计引进市外（国内）资金265亿元，是“九五”时期的9．8倍。与俄蒙经贸合作转型升级，境外资源开发、境内加工成效明显，鲁能、新鑫、大庆油田公司等企业取得俄蒙资源开发权，满洲里进口产品加工规模快速扩大。

（三）基础设施条件进一步改善

生态建设方面，以构筑祖国北疆生态安全屏障和增强可持续发展能力为目标，加大了生态环境保护和治理力度，实施了草原保护与建设、防沙治沙、退耕还林还草、天然林保护、生态公益林保护等重点工程，累计投入生态建设资金15亿元，生态环境得到进一步改善；水利建设累计争取国家投资59．4亿元，建成了尼尔基大型水利枢纽工程。交通建设方面，五年公路建设总投资完成76亿元，是“九五”期间投资总额的6倍，修建及改建公路里程2908公里，实现了旗市全部通油路；开工建设了伊敏———伊尔施铁路，开通了满洲里机场，实施了海拉尔机场改扩建工程。城市建设方面，各旗市区所在地城镇面貌得到较大改善，涌现出了一批各具特色的小城镇；启动了一大批城镇给排水、垃圾处理、供热供气、出口路改造等基础设施建设项目；基本实现了市域范围内电网统一，进一步完善了通信基础设施。加快了信息化步伐，呼伦贝尔门户网站等政务网站建设得到加强，网上政务事务公开、打造“网上阳光政府”取得良好效果，地税、工商基础信息共享互联互通试点工程取得初步成效。

（四）城乡人民生活水平稳步提高

全市城镇居民人均可支配收入达到8228元，比2000年增长75．8％，年均增长12．0％；农牧民人均纯收入达到3202元，比2000年增长46．1％，年均增长7．9％。城乡居民储蓄存款余额达到228．9亿元，比2000年增长77．7％，年均增长12．2％，社会消费品零售总额达到108．12亿元，比2000年增长95．0％，年均增长14．3％。城乡居民消费结构发生显著变化，教育、卫生、文化消费支出大幅度增长。就业再就业工作进一步加强，累计实现就业16．8万人，下岗失业人员再就业9．6万人，失业率控制在4．7％以内。社会保障体系不断完善，建立健全了国有企业下岗职工基本生活保障、失业保险和城镇居民最低生活保障制度，社会保险覆盖面和保障水平不断提高；社会福利和困难群体救助工作得到加强，农村牧区贫困人口大幅度减少，累计解决了62万贫困人口的温饱问题。

（五）各项社会事业和民主法制建设得到加强

深入实施科教兴市战略，科技成果向现实生产力转化进程加快。普及九年义务教育取得突出成就，教育基础设施和教学条件不断改善；中等职业教育资源配置得到优化，办学规模进一步扩大；高等教育结构调整、资源重组工作进展顺利，呼伦贝尔学院完成了与七所院校的合并，成功晋升为本科院校；民族教育办学条件得到改善，“两主一公”的民族教育模式基本实现。

医疗卫生条件进一步改善，初步建立起了重大疫情等突发公共卫生事件应急机制。人口继续保持较低增长速度，低生育水平得以稳定。民族文化大市建设取得积极进展，广播电视、新闻出版、文学艺术和社会科学事业繁荣活跃。群众性精神文明创建活动蓬勃开展，公民道德素质明显提高。坚持依法治市，积极推进政府职能转变，提高了决策民主化、科学化水平。全市各级政府认真执行人民代表大会及其常委会的各项决议，主动接受人大法律监督、政协民主监督和人民群众社会监督。审计监督力度进一步加大，廉政建设和反腐败斗争不断加强。健全了群众来信来访工作机制，妥善处理群众反映强烈的突出问题和群体性上访事件。普法教育广泛开展，社会治安综合治理工作得到加强，严厉打击各种刑事犯罪专项斗争成效显著，维护了社会稳定。侨务和对台工作取得新的进展。国防教育和国防后备力量建设得到加强，军警民共建和“双拥”工作深入开展，荣获全国“双拥”模范城称号。党的民族、宗教政策进一步落实，民族团结、社会稳定、边疆安宁的局面得到巩固和加强。

各位代表，2005年是“十五”的最后一年。全市上下坚持以科学发展观为指导，以改革开放为动力，以项目建设为重点，经济建设和社会事业走上了又快又好发展的轨道。一是经济发展速度和质量同步提高，经济总量迈上新台阶。地区生产总值增速达到22％，连续两年保持两位数2字头增长，增速排在全区第五位，在东四盟市排在第一位。财政收入比上年增加8．04亿元，比年初预算超收2．5亿元；增速达到29％，比地区生产总值增速高出7个百分点；财政收入占地区生产总值比重达到11．2％，比上年提高0．6个百分点。二是工业化进程加快，农牧业结构调整取得新进展。全部工业增加值达到69．64亿元，增长33．2％；工业增加值占地区生产总值比重达到21．8％，比上年提高1．7个百分点；能源重化工业、农畜产品加工业、进出口产品加工业已成为经济发展的主要支撑力量。全市牧业年度牲畜总量达到1482．4万头只，其中农区牲畜总量达到876．9万头（只），占全市牲畜总量的59．2％，比上年提高8．4个百分点；粮食产量达到55．6亿斤，其中玉米产量达到15．4亿斤，“为养而种”正在成为广大农民的自觉行动。三是项目建设决战年成效显著，经济发展的后劲明显增强。通过招商引资的强力拉动，全社会固定资产投资完成192．6亿元，比上年增加56．85亿元，其中地方城镇固定资产投资完成175．1亿元，比上年增长48．2％；全年完成投资超亿元的项目25项。建成投产了海拉尔热电2×5万千瓦、宝日希勒千万吨煤矿改造及快速装车系统、中集集装箱板、尼尔基水利枢纽工程、牙克石———海拉尔一级公路、天骄生态园等一批重点项目。开工建设了伊敏二期2×60万千瓦、雀巢日处理700吨乳制品、海农集团30万吨油菜籽加工、海拉尔机场候机楼、海———满一级公路、满洲里香格里拉酒店、海拉尔华联商厦等一批重点项目。同时，一大批电力、煤化工、冶金、农畜产品加工、旅游、商贸、交通、城建等重大项目具备了开工条件。四是对外开放迈出新步伐，深化改革取得新进展。紧紧抓住对俄蒙开放升级和东北振兴的机遇，全面扩大了对外开放。与东北合作有了新进展，我市被列入国家振兴东北等老工业基地规划范围，2005年引进的项目，来自东北的占一半以上。与长三角、珠三角、环渤海等地区合作得到加强，地区间、企业间的联系进一步加深，寻求在我市投资合作者逐步增加。全市进出口总额完成16亿美元，增长15．4％；口岸过货量达到1764万吨，增长25．2％。开发利用俄蒙资源取得明显进展，对俄罗斯别列佐夫铁矿开发、森林开发、木材加工和对蒙古国铅锌矿开发、石油开发等项目顺利实施。满洲里进口木材加工园区快速发展壮大，年木材加工能力达到了300万立方米。农垦系统“双退出”工作平稳完成，农垦集团朝着现代企业制度迈出了重要一步，恩和牧场实行了属地管理。神华集团重组宝日希勒煤业公司，建设大型煤电油化基地，增强了国有企业经济实力和发展活力。金融体制改革稳步推进，金融机构对地方经济发展支持力度不断加大，中小企业融资难、贷款难问题得到有效缓解。税费改革进一步深入，阿荣旗乡镇机构改革试点工作顺利完成，为在全市推开积累了经验。五是人民群众得到更多实惠，各项社会事业全面发展。城镇居民生活水平进一步提高，城镇居民人均可支配收入比上年增长15．1％，增速在东四盟市排在第一位；城镇居民最低生活保障人均月补差标准达到63．7元，比上年增加10．1元，兑现了政府年初作出的承诺；新增就业32755人，其中下岗再就业18176人，“4050”人员再就业4330人，解决零就业家庭一人就业问题成效突出，全市登记在册的2032户零就业家庭中有2449名成员实现就业，全市城镇登记失业率控制在4．36％以内。农牧民增收渠道进一步拓宽，全部减免农业税使广大农牧民直接受益1．7亿元；政府支农支牧力度继续加大，支持“三农三牧”资金达到51880万元，比上年增加8731万元，增长20．2％；农牧民人均纯收入比上年增长19．4％，增速在全区排第二位，在东四盟市排第一位。科技、教育、文化、体育、医疗卫生、人口计划生育、广播电视、新闻出版等事业获得长足进步。安全生产工作得到加强，全年没有发生特大安全生产事故，受到自治区党委、政府的表彰。扎实开展了“信访攻坚年”活动，解决了一大批信访老大难问题，涉法案件、城市拆迁、农村牧区土地草场纠纷等信访问题明显减少。清理拖欠工程款力度加大，全年清理拖欠工程款5．1亿元，其中清理政府拖欠工程款3．4亿元。顺利完成了呼伦贝尔市一届人大五次会议确定的各项奋斗目标，为“十五”计划画上了圆满句号。

五年来，我们在实践和发展中，积累了许多宝贵经验。主要是：坚持重点突破为前提，进一步明晰了发展思路，解放思想，抢抓机遇，全力推进工业强市战略，加快工业化、农牧业产业化、城镇化进程，有力促进了各产业快速协调发展；坚持改革开放为动力，以改革开放牵动经济工作全局，不断优化经济发展环境，引进大企业，建设大基地，大范围、宽领域配置生产要素，不断提高经济运行质量；坚持生态立市为基础，始终坚持绝不以牺牲环境换取发展的思想，使经济发展与资源、环境相协调；坚持改善民生为目标，努力提高各族人民群众的生活水平，使人民群众共享经济发展的成果；坚持转变作风为保障，抓住各级领导班子和领导干部队伍建设这一关键，强力推进以经济建设为中心的各项工作任务的落实。

各位代表，“十五”成就的取得，特别是2005年各项工作成绩的取得，是自治区党委、政府和市委正确领导、科学决策的结果，是四大班子精诚团结、共同努力的结果，是全市各级干部群众攻坚克难、真抓实干的结果，是社会各界大力支持、团结奋斗的结果。在这里，我代表市人民政府，向在各个领域和岗位上辛勤工作的全市各族干部群众，向曾经为呼伦贝尔建设和发展做出贡献的老领导、老同志，向在呼伦贝尔投资创业的国内外企业家们，向关心和支持呼伦贝尔建设与发展的各界朋友，表示衷心的感谢和崇高的敬意！

过去五年取得的成就是巨大的，经验是可贵的，为我市“十一五”时期实现又快又好发展奠定了坚实基础。但也必须清醒地看到经济社会发展中存在一些不容忽视的困难和问题。主要表现在：生产力不发达，经济发展速度相对滞后，产业结构不尽合理，传统产业多，现代产业少，尤其是农牧业基础设施薄弱，靠天吃饭的状况没有从根本上扭转，农牧民增收困难；区域经济发展不平衡，旗市区间发展的速度和质量差异较大，经济一体化进程亟待快速推进，森工、煤炭、农垦等大企业的改革发展有待进一步深化；经济发展的外向度偏低，对外开放程度与对外开放优势不相称，两种资源、两个市场综合开发利用水平不高；产业集群化发展不够，产业链条不长，自主创新能力较弱，产业技术水平较低，经济增长方式比较粗放。同时，需要说明的是：由于地区生产总值统计方法和基数调整，我市未能完成2005年地区生产总值330亿元的目标；由于受国家宏观调控和核准程序影响，部分原定开工的项目推迟到今年。解决这些困难与问题，根本出路在于加快发展。发展滞后是我市面临的最为紧迫、最为重要的问题，在科学发展中崛起，是自治区对我们的明确要求，也是全市人民的迫切愿望，更是本届政府和各级干部肩负的历史责任。

二、“十一五”时期的奋斗目标和主要任务

“十一五”时期，是我市全面落实科学发展观、推进新型工业化进程、实现富民强市兴边的关键时期。按照把我市建设成为东北乃至全国重要的能源战略高地、绿色食品基地、休闲旅游胜地、北疆开放龙头、自然生态屏障、平安和谐家园的总体发展定位，“十一五”时期全市经济社会发展的总体要求是：以邓小平理论和“三个代表”重要思想为指导，以科学发展观统揽全局，以富民强市兴边和全面建设小康社会为目标，以改革开放为动力，以生态立市、工业强市、旅贸活市、科教兴市、文化名市为发展模式，抓住综合战略机遇，强力推进工业化、农牧业产业化、城镇化进程，大力发展循环经济，促进经济增长方式转变，优化产业结构，加快建设“三大基地”，积极构筑“七大产业集群”，构建和谐社会，努力推进经济社会可持续、高质量、跨越式发展，为实现呼伦贝尔在科学发展中崛起奠定坚实基础。“十一五”时期全市经济社会发展主要目标是：地区生产总值年均增长20％，达到800亿元；人均生产总值年均增长19．1％，达到3470美元；固定资产投资年均增长25％，达到595亿元；工业增加值年均增长35％，达到312亿元，财政收入年均增长22．9％，达到100亿元；城镇居民人均可支配收入年均增长13％，达到15200元；农牧民人均纯收入年均增长10．4％，达到5250元。生态环境质量整体好转，循环经济发展初见成效，资源利用率显著提高，单位生产总值能耗比“十五”末降低10％。经济结构和产业结构更加合理，工业化水平、城镇化水平、农牧业产业化水平、经济外向度有较大幅度提高。各类教育继续发展，就业形势比较稳定，社会保障体系比较健全，农村牧区面貌明显改善，民主法制建设和精神文明建设取得新进展，社会更加和谐。

实现上述目标，机遇和挑战并存。我们必须紧紧抓住西部大开发深入实施、东北振兴加快推进、北疆开放战略升级、东部盟市加快发展、民族地区备受重视等综合机遇，立足于资源、区位、产业等比较优势，按照科学发展观的要求，转变发展观念，创新发展模式，提高发展质量，增强竞争优势。在具体工作中要把握好以下原则：必须坚持快速发展的原则，按照“十一五”发展规划和目前已经凸显的发展态势，一些重点工业项目在“十一五”期间将陆续建成投产，带动经济总量实现成倍增长或者几倍增长都是可能的，快速增长将成为“十一五”时期呼伦贝尔经济发展的一个重要标志；必须坚持理性发展的原则，经过几年来的不断探索，呼伦贝尔的发展目标和思路以及发展措施已经非常清晰，“重点突出、有进有退”的理性发展道路已经成为全市上下的共识；必须坚持协调发展的原则，呼伦贝尔作为资源大市，在加快发展的过程中，要保证经济、政治、文化建设的各个环节、各个方面相协调，最现实、最基本的任务是要切实转变经济增长方式，提高经济增长的质量和效益，努力实现速度与结构、质量、效益相统一，经济发展和人口、资源、环境相协调，保持和增强发展的可持续性；必须坚持全面发展的原则，以经济建设为中心，全面推进经济、政治、文化和社会建设，切实保障人民群众的经济、政治和文化权益，让发展的成果惠及全市人民；必须坚持共同发展的原则，紧紧抓住国家更加重视少数民族和民族地区发展的机遇，切实解决好少数民族和自治旗、民族乡的发展问题，解决好农区、牧区、林区、垦区、矿区共同发展问题，实现不同人群的共同发展。“十一五”期间，我们要重点做好以下工作。

（一）围绕建设经济强市，全力推动优势特色产业发展壮大

依靠能源重化工业突破，加快推进工业化进程。依托资源禀赋和产业基础，以重点项目建设为切入点，做大做强优势特色产业。重点发展煤炭、电力、化工、冶金、绿色农畜产品加工、进出口产品加工等优势产业，积极培育新型建材、生物制药等特色产业。坚持实施产业集聚战略，大力发展园区经济和基地经济，建设形成伊敏、大雁、宝日希勒、扎赉诺尔等能源重化工业基地。加强地质勘查工作，尽快摸清家底，为发展能源重化工业提供资源保障。进一步完善土地、矿业权市场体系，提升市场对资源的配置功能。

依靠农牧业产业化突破，加快推进新农村牧区建设。按照建设“生产发展、生活宽裕、乡风文明、村容整洁、管理民主”的社会主义新农村要求，坚持“工业反哺农牧业、城市支持农村牧区”的发展思路，坚持“多予、少取、放活”的方针，合理制定发展规划，加强分类指导，抓好试点示范，增强工业对农牧林业发展的带动作用，提高农牧民和林区群众的持续增收能力，加快农牧林区基础设施建设和各项社会事业发展，努力改变农牧林区的落后面貌。

依靠旅游业突破，加快推进第三产业快速发展。重点加强区域旅游通道、重点旅游景区基础设施建设，构建旅游产业集群，全面提升旅游业的产业素质和竞争力。加快商贸流通、运输邮政、餐饮等传统服务业的提升改造力度，提高市场化、产业化、规模化水平。加快发展金融、信息、会展、房地产和社区、物流等现代服务业。

（二）围绕增强经济社会发展活力，全面深化改革和扩大开放

推进体制机制创新。适应经济体制和经济增长方式根本性转变的要求，加快煤炭、森工、农垦、市属林业六局等国有大企业改革步伐，抓好主辅分离、辅业改制和分流安置富余人员工作，培育产权清晰、权责明确、政企分开、管理科学、主业突出、具有竞争优势的大型企业集团。坚持长期稳定家庭承包经营制度，进一步完善土地、草牧场使用权流转制度。科学整合乡镇资源，大力推进乡镇机构改革。深化投资管理体制和财政管理体制改革，清理整顿不合理收费，实施“收费阳光工程”。完善价格监测和预警制度，创新价格与收费监管方式，规范市场价格秩序。积极稳妥地深化事业单位改革，推进政事分开、管办分开和多元投入。放宽市场准入，加大政策和资金扶持力度，建立和完善中小企业担保体系，营造适宜非公有制经济成长的发展氛围。

努力扩大对外开放。实施中俄蒙毗邻地区“大金三角”战略，加快与俄蒙经济技术合作步伐，推进境外资源过埠转化和开发利用，带动口岸经济实现快速发展。力争“十一五”末，全市进出口总额达到32亿美元，口岸通关能力达到6700万吨、1200万人次。积极参与东北振兴，借力加快发展。主动承接环渤海经济圈、长三角、珠三角等经济发达地区产业转移，千方百计促进资金、技术、人才等生产要素向我市汇聚。“十一五”期间，全市引进市外（国内）资金累计达到1200亿元以上，实际利用外资累计达到1．3亿美元以上。

（三）围绕城乡统筹发展，着力推进城镇建设和生态建设

大力提高城镇化水平。坚持“因地制宜、分类指导、民营主体、特色突出”的原则，培育壮大旗市所在地城镇主导产业，促进旗市区经济加快发展。努力把海拉尔建成全市具有较强辐射和聚集功能的区域性核心区，把满洲里建成国际性综合口岸城市，把牙克石建成辐射林区的商贸物流和生产生活物资供应中心，把扎兰屯建成滨洲线上辐射岭东和黑龙江毗邻地区的绿色农畜产品加工基地，把各旗市所在地城镇建成具有较强辐射和带动能力的经济中心。完善城镇功能，高起点做好城镇发展规划，高标准推进城镇建设，高水平抓好城镇管理。加快推进城乡户籍、土地、劳动就业和社会保障制度改革，积极创造有利于农牧林区人口向城镇转移的外部环境。

强化生态环境建设。全面推进生态市建设，确定生态立市理念，坚持美丽与发展双赢，绝不走先发展后治理的弯路。加强污染防治，严格控制污染物排放总量，认真执行环境准入制度，改善重点区域、重点流域环境质量。加强生态保护与建设，有效遏制生态恶化趋势，全面提升呼伦贝尔草原、森林、河湖、湿地的生态功能。加强资源的合理开发和有效保护，积极发展集约化生产，提高资源利用效率和综合利用水平。发展循环经济，推行清洁生产和建筑节能工作，努力建设资源节约型社会和环境友好型社会。巩固生态示范区建设成果，“十一五”期间，50％以上旗市达到全国生态县标准，海拉尔区争创国家环保模范城市。

（四）围绕提高人民生活水平，大力推进各项社会事业发展

努力提高城乡居民收入。加强就业和再就业工作，努力创造更多就业机会，提高城镇居民特别是低收入人群的收入水平。加大企业职工增资力度，保证职工工资收入与企业效益和地方财政收入同步增长。积极落实国家有关工资调整政策，适时适度提高机关公务员和事业单位职工工资收入。调整优化农村牧区经济结构，大力发展农牧业产业化经营，加快发展非农产业，积极推动劳务输出，促进农牧民拓宽增收渠道，提高农牧民持续稳定增收能力。

加强社会保障体系建设。建立完善城镇职工基本养老保险、失业保险、基本医疗保险、工伤保险、生育保险、农村牧区养老保险和城市最低生活保障制度，完善农村牧区社会救济制度，形成健全完善的社会保障体系。加快将非公有制企业、有条件参保的灵活就业人员、进城务工人员纳入社会保障覆盖范围，基本实现城镇各类从业人员平等享有社会保障。逐步建立和推行农村牧区最低生活保障和医疗救助制度，构建和完善农村牧区综合救助体系。加大城镇和农村牧区弱势群体的救助力度，不断提高社会救济和社会福利的投入。

深入实施科教兴市战略。加快科技攻关、成果转化和产业化进程，不断提高全民科技素质和自主创新能力。深化教育体制改革，全面实施素质教育，加快教育结构调整，优化教育资源配置，推动各级各类教育均衡协调发展，不断满足广大人民群众对各级各类教育的需求。大力发展职业教育，加快培养科技创新及先进制造业、现代服务业和农牧业发展急需的技能型人才。推进人才强市战略，牢固树立“人才资源是第一资源”和“关键在人才”的理念，不断完善人才培养引进政策，培养富有创新能力的专业人才，全面实施各项人才工程，加快人才净流入区建设，形成各类人才竞相涌现、各种创造力竞相迸发的人才成长创业环境。

统筹发展各项社会事业。积极推进民族文化大市建设，深化文化管理体制改革，加快文化产业化发展步伐，提高广播电视、新闻出版工作水平。加强人口和计划生育工作，稳定低生育水平，提高人口素质，改善人口结构。以农村牧区为重点，加快公共卫生体系建设，普及新型农村牧区合作医疗，完善突发公共卫生事件应急机制，加强对重大传染性疾病的群防群控，深化城镇医疗卫生体制改革，逐步解决群众“看病难、看病贵”问题。积极开展全民健身活动，大力发展体育事业，提高体育竞技水平和公民的身体素质。

创建安全稳定社会环境。统筹兼顾不同社会利益群体，坚决制止和纠正损害群众利益行为，维护社会公平。积极预防和妥善处置群体性事件，健全大防控、大调解机制，加大综合治理力度，建设“安全稳定呼伦贝尔”。加强宗教事务管理，积极引导宗教与社会主义社会相适应。落实安全生产责任制，预防和减少重特大事故发生。

三、2006年经济和社会发展的主要任务

2006年是实施“十一五”规划的起步之年，做好今年各项工作，保持经济社会的良好发展势头，确保“十一五”开好局、起好步，对于呼伦贝尔在科学发展中崛起具有重要意义。今年我市经济社会发展主要预期目标是：生产总值增长20％，达到390亿元；城镇单位固定资产投资增长29．6％，达到227亿元；财政收入增长20．1％，达到42．88亿元；城镇居民人均可支配收入增长13％，达到9300元；农牧民人均纯收入增长12．4％，达到3600元；城镇登记失业率控制在4．3％以内，人口自然增长率控制在5．4‰以内。

完成上述目标，必须认真做好以下重点工作。

（一）以推进新型工业化为重点，加速扩张工业经济的总量

巩固项目建设决战年成果，举全市之力，全力抓好86个投资超2000万元的重点项目建设，特别是要搞好54个投资超亿元的重大项目建设，完成工业投资117亿元。重点抓好伊敏二期2×60万千瓦、深能源2×20万千瓦、国华风电2×5万千瓦等续建项目建设，开工建设海拉尔2×20万千瓦、360万千瓦招标外送电源、50万伏直流输电通道等项目。加快实施宝日希勒1000万吨露天矿、鲁能集团五牧场河东500万吨煤矿、外送电源点配套煤炭开发等项目建设。支持大庆呼伦贝尔分公司进一步扩大产能，石油产量争取达到50万吨以上。开工建设神华宝日希勒180万吨甲醇、52万吨丙烯项目，西洋大雁40万吨甲醇项目，香港金新化工80万吨尿素、52万吨合成氨等化工和冶金项目建设。加快雀巢奶粉加工、喜洋洋肉类加工、海农集团油菜籽加工、扎兰屯玉米酒精、阿荣旗中敖30万吨燃料乙醇和肉类加工等项目建设。抓好中集集装箱板二期、兴安玖龙二期、联发进口木材深加工等木材加工项目建设。积极推进企业股权多元化步伐，加快资产重组工作，搞好企业的引资扩张，支持优势企业做强做大，推进停产、半停产企业盘活资产，努力形成新的增长点。加大对企业技术创新和技术改造的政策支持力度，推动重点行业、重点企业、重点产品的技术改造，努力培育自治区和国家级知名企业、名牌产品。抓好基地和园区规划设计，合理利用土地等资源，提高单位面积产出效益；加快完善工业园区水、电、路、气、排污等配套设施，为入驻企业提供良好的发展平台。加强工业生产调度，及时解决企业在生产经营过程中遇到的运输、资金、电力等方面的问题，全力保证企业满负荷生产。

（二）以提高农牧民收入为核心，全力推进新农村牧区建设

优化调整农牧业经济结构。大力发展农区畜牧业，突出发展奶牛饲养和牛羊育肥业，走种养业以养殖为主、养殖业以乳肉为主的发展道路。力争畜牧业产值占第一产业的比重达到45％，比2005年提高5个百分点。积极调整种植业产品结构，在稳定粮食生产的基础上，大力发展优良品种、特色品种，加快发展优质、专用、无公害、绿色和有机食品，粮经饲比例达到79．5∶15．7∶4．8。抓好农牧林业品牌开发，打好“绿色牌”、“特色牌”，提高农牧业比较效益。

大力推进农牧业产业化经营。搞好农畜产品加工业布局调整，扶持重点龙头企业扩大规模，鼓励企业引进、开发新产品，提高加工增值水平，农畜产品加工转化率力争达到50％。重点扶持雀巢、伊利、光明、三元、洛娃、元盛、淳江、鹤声等优势企业做大规模，提高加工深度，延伸产业链条，提高产品档次。鼓励龙头企业独立或联合发展多种形式的原材料生产基地，大力发展订单农牧业，引导龙头企业与农牧民在产业化经营中逐步形成互惠互利、共担风险、共同发展的联结机制。积极支持农村牧区各类经济合作组织发展，提高农牧民的组织化程度。

认真落实各项惠农支农政策。落实各项补贴政策，巩固税费改革成果，坚决遏制各种乱收费、乱摊派、乱罚款，防止农牧民负担反弹。建立健全质量标准体系，加强质量安全检测监督，保证农畜产品质量安全。加强动植物疫病防控体系建设，高度重视禽流感、口蹄疫等疫病的防控工作，防疫密度要达到100％。继续抓好科学种养适用技术的推广，搞好农牧民技术培训，努力转变落后的农牧业生产经营方式。积极发展劳务经济，鼓励和引导农牧民开展劳务输出，增加劳务收入。

加强农村牧区公共服务体系建设。切实抓住国家加大对农村牧区公共服务体系建设投入的大好机遇，全面加强农牧业基础设施建设，着力改变农牧业生产条件落后的现状，提高农牧业生产的现代化水平。切实加强农村牧区文化、教育、卫生、社会保障工作，提高农牧民的生活质量。

抓好新农村牧区建设规划和试点工作。按照中央建设社会主义新农村的总体要求，以“发展新产业、建设新民居、创建新机制、培养新农牧民、塑造新风貌、建好村嘎查班子”为工作重点，认真抓好新农村牧区建设总体规划和调研试点工作，为全市推进新农村牧区建设积累经验。

（三）以扩大消费需求为导向，积极推进第三产业快速发展

认真落实自治区和我市扶持第三产业发展的优惠政策，加快第三产业开放步伐，大力发展旅游休闲、商业贸易、房地产开发等生活型服务业和现代物流、金融保险、研发咨询等生产型服务业，加快交通、通讯、教育、文化、体育等社会事业市场化运作进程，促进社会消费多元化，依靠服务业拓展鼓励消费增长。尤其要进一步精心培育发展旅游业。依托草原、森林、河湖、冰雪、口岸、历史、民俗等优势旅游资源，推进八大类、26个重点旅游招商项目建设，精心策划和推出旅游线路和旅游产品，抓好星级酒店、旅游通道建设，着力解决好景区景点老化、旅游产品单一、旅游服务配套设施不完善等问题，打造旅游精品亮点，全面提升旅游产业的整体竞争力。以举办各种节庆活动和旅游宣传促销为载体，加强与俄蒙、东北、东部盟市以及北京、上海、广州、哈尔滨等主要客源地旅游企业、新闻媒体的交流与合作，促进旅游市场一体化建设，不断提高呼伦贝尔旅游知名度。全年旅游业总收入达到51．6亿元，增长25％。

（四）以增强宏观调控能力为切入点，全面做好财税金融工作。

加强税收征管，积极培植税源，挖掘税费收入潜力，提高财政收入质量，确保财政收入持续快速稳定增长。积极争取上级财政支持，合理安排本级预算，优化财政支出结构。积极发挥财政调控经济职能，推动重点区域、重点产业、重点项目加快发展和建设步伐。加快公共财政体系建设，扩大对社会保障、义务教育、公共卫生、农牧民增收、农牧业产业化和农村牧区基础设施建设的投入。加强社会信用体系建设，建立健全中小企业投资担保体系。积极开展与国家开发银行、信托机构和市内金融机构的融资合作，努力拓宽融资渠道。继续推进农村信用社改革，加快组建农村合作银行，提高为“三农三牧”服务水平。

（五）以招商引资为突破口，大力提高对外开放水平和档次

多渠道全方位招商引资。充分发挥优势资源、优势产业的积聚效应，全面开放交通、城建、水利、服务贸易、教育、旅游等投资领域，积极优化和推介呼伦贝尔的投资环境，大力吸引外来资金进入我市投资领域。强化政府引导，突出市场运作，创新招商方式，通过专业招商、委托招商、网络招商、以商引商等多种形式，确定重点地区、重点项目、重点企业，主动上门对接，积极引进大公司、大财团和知名企业入驻我市。建立健全招商引资激励机制。继续抓好投资软环境建设，做到爱商、亲商、富商、留商，促其增资扩产，影响和带动新的投资者；营造政策稳定透明、办事诚信高效的投资环境，使呼伦贝尔真正成为吸引外来投资的热土。力争全年引进市外资金137亿元，实际利用外资0．36亿美元。

多层次扩大对外经贸合作。发挥毗邻俄蒙、口岸众多两大优势，以举办中俄毗邻地区“俄罗斯年”启动仪式、“中俄蒙边境区域合作论坛”和“中俄矿产资源开发对接会”为契机，搭建与俄蒙交流合作平台，推进与俄蒙的多领域合作。加快转变对外贸易增长方式，优化出口商品结构，支持地产品、轻工产品、机电产品出口，鼓励企业进口木材、矿产、石油等紧缺原材料，积极发展进口产品加工贸易。加快完善“大通关”体系，完善满洲里互市贸易区、进出口产品加工区，提升黑山头、室韦、阿日哈沙特、额布都格口岸功能，加快口岸经济发展，力争全年口岸过货量达到2000万吨。深入实施“走出去”战略，支持企业到境外投资兴业、开发资源、拓展国际市场。积极参与东北振兴，在规划、政策、通道、项目等方面全面对接。主动承接东部沿海发达地区产业转移，多形式开展与东北地区、沿海地区的战略合作，发展壮大外向型经济。

大力发展非公有制经济。积极营造适宜非公有制经济发展壮大的良好环境，使非公有制企业经营者在社会上有声誉、政治上有地位、经济上有发展。完善和落实推进非公有制经济发展的相关政策，加大对非公有制经济的扶持力度，进一步放开经营范围，并在立项审批、信贷投放等方面与国有经济实行同等待遇。鼓励和支持非公有制企业购买、控股、参股、租赁国有或集体企业，实现低成本扩张。要强化政府的引导和服务，搞好宏观发展规划，在产业指导、政策咨询、信息提供等方面，为非公有制经济发展提供规范的法制环境、良好的信用环境、完善的服务环境。

（六）以完善区域功能为目标，全面加大基础设施建设力度

搞好交通建设。公路建设完成投资20亿元以上，建设公路里程1226公里以上。抓好海拉尔———满洲里一级公路及子项目、根河———白鹿岛、谢尔塔拉———特尼河、大杨树———古里乡等公路项目建设。加快实施伊敏———伊尔施、滨洲复线铁路工程，积极争取建设地方、合资铁路。加快民航海拉尔东山机场改（扩）建项目建设，启动满洲里机场二期工程。加强口岸基础设施建设，重点做好室韦、黑山头口岸铁路建设前期准备工作。

抓好城乡建设。全面启动呼伦贝尔中心城新区建设，抓好城关镇、中心城镇给排水、垃圾处理、城镇道路改造、园林绿化等项目建设，搞好城乡结合部、出口路、平房区和城中村的改造。抓住国家更加关注社会主义新农村建设、政策资金向农村倾斜的有利时机，加大财政对农村牧区公共设施建设投入的引导，推进农村牧区道路、饮水、电网、通讯等基础设施建设。加强生态建设。大力实施退耕还林还草、退牧还草、防沙治沙、水土保持治理、生态移民、天保工程和重点公益林保护工程，完成退耕100万亩、人工造林及封沙育林20万亩、草原禁牧400万亩、休牧3600万亩，严禁超载过牧现象发生。加快推进毕拉河口、扎罗木得、红花尔基水利枢纽工程项目前期工作，做好尼尔基水库下游灌区规划，抓好安全饮水、节水灌溉、水土保持生态建设、水库除险、国际界河护岸等工程建设。

（七）以解决群众切身利益为根本，加快各项社会事业发展

努力提高人民收入和社会保障水平。进一步落实各项扶持政策，加大劳动密集型产业岗位和公益性岗位的开发力度，加强劳动技能培训，以解决困难群体就业为重点，切实做好就业和再就业工作，全年实现新增就业2．5万人。进一步做好养老、失业、医疗、工伤、生育保险和住房公积金扩面征缴工作，提高城市最低生活保障人均月补差标准，启动农村牧区最低生活保障制度。推进廉租房和经济适用住房建设，改善城镇中低收入家庭住房条件。认真贯彻《公务员法》，适当提高公务员工资。加大清理拖欠工程款和农民工工资力度，确保施工企业和农民工的合法权益不受侵犯。

全面推进各项社会事业快速发展。大力推进科技进步，抓好科技创新能力建设，加强科普工作，加快科技示范工程建设，增强科技对经济和社会发展的支撑能力。加快发展教育事业，不断加大教育特别是农村牧区义务教育的投入，抓好“两基”攻坚，整合中等职业教育资源，提高高等教育教学质量。进一步繁荣文化事业，加大文艺精品创作力度，大力发展文化产业，加快培育文化娱乐市场，推动民族文化大市建设。加强信息化基础建设，整合电子政务资源，不断推进网上政务事务公开和网上办事。

推进疾病预防控制、医疗救治和卫生监督体系建设，积极推广新型农村牧区合作医疗，加强医疗单位服务价格和成本要素的市场检测和规范管理，加强蒙医、蒙药的研究、开发和推广工作，改善各族群众的就医条件。加强食品、药品和餐饮卫生监管，确保人民群众身体健康和生命安全。认真落实计划生育政策，稳定低生育水平。加强广播电视、新闻出版工作，不断提高新闻媒体的覆盖率。积极发展体育事业。依法管理民族宗教事务，重视老龄人口和青少年管理工作。加强精神文明建设，大力弘扬爱国主义和团结奋进、开拓创新、文明诚信、昂扬向上的时代精神，全面提高公民的思想道德素质。深入开展“双拥”工作，争创全国“双拥”模范城两连冠。进一步做好扶贫、统计、救灾、外事、人防、侨务和对外联络等工作。

切实维护社会稳定。高度重视人民群众来信来访和人民调解工作，认真落实信访工作领导责任制，关心群众疾苦，及时化解矛盾纠纷。建立完善群体性事件预警、应急、处置机制，提高保障公共安全和处置突发事件的能力。以创建“安全稳定呼伦贝尔”为目标，加强社会治安综合治理，依法严厉打击各种刑事犯罪和敌对势力的分裂破坏活动。建立健全国防动员体系，支持国防重点工程建设。认真落实各项安全生产责任和监管措施，切实抓好煤矿、交通、森林草原防火、城镇消防和高危行业的安全生产，有效遏制重特大事故，确保人民群众的生命财产安全。

今年，我市要在认真落实自治区政府提出的为人民群众办好6件实事的基础上，积极创造条件，为我市人民群众再办6件实事。

一是实施农村牧区饮水安全工程，开工建设乡镇自来水工程16处，打机电井21眼，解决3．2万人、4．15万头（只）牲畜饮水安全问题。

二是对贫困家庭寄宿学生发放生活费补助，每生每年补助300元，补助面不低于全市全部农村、牧区寄宿生的40％。实施民族学校少数民族（三少民族、蒙古族、俄罗斯族）贫困家庭住宿生助学金制度，助学金标准为小学生每月60元、中学生每月80元，享受助学金的范围不低于少数民族住宿生总数的50％。

三是进一步整合牧区中小学资源，将牧区初中由24所调整为17所、小学53所调整为24所，彻底实行“两主一公”的办学模式。

四是加强体育基础设施建设，建设群众性体育活动场馆。开工建设建筑面积为18000平方米的呼伦贝尔市体育馆，其它旗市也要加强群众性体育设施建设。

五是实施城市道路建设改造工程，重点抓好巷道的新建、改造和附属设施建设，改善居民的出行环境。全市改造90条街巷，总面积60万平方米，安装路灯1600盏。

六是继续开展“蓝天工程”建设，进一步解决冬季煤烟大气污染问题。重点解决海拉尔区煤烟的空气污染问题，其它旗市也要创造条件，解决冬季煤烟污染问题。

（八）以提高行政效能为中心，全面加强政府自身建设

切实提高行政效能。强化服务意识，切实把政府职能转变到经济调节、市场监管、社会管理和公共服务上来，全面提高政府为基层、企业、投资者的服务水平。强化效能意识，推进政府权力运行的公开化、规范化和制度化，加强政府各部门之间的分工协作，简化办事程序，提高工作效率，树立一盘棋思想，坚决杜绝推诿扯皮现象发生。

全面推进依法行政。贯彻落实国务院《全面推行依法行政实施纲要》，按照法定权限和程序行使权力，履行法定职责，依法处理社会经济事务，确保法律法规的正确实施。深化行政执法体制和行政审批制度改革，规范行政执法、行政审批行为。完善监督机制，形成监督合力，加强对行政权力的监督，推进法治政府、责任政府和服务型政府建设。以素质建设、能力建设为重点，认真落实《公务员法》，坚持从严治政，健全教育、制度、监督体系，全面提高公务员队伍素质。

自觉接受各界监督。严格执行法律法规，自觉接受市人大及其常委会的法律监督和工作监督，接受市政协的民主监督，加强与各民主党派、工商联、人民团体和无党派代表人士的联系。认真执行市人大议案决议，办理好人民代表建议和政协提案。接受新闻舆论和社会公众监督，重视人民群众通过行政复议、行政诉讼等法定渠道，对政府机关及其工作人员的监督。加强重点领域、重点部门和重点资金的审计监督，及时解决和处理审计中发现的问题。办好市府热线，认真解决群众反映的热点难点问题。

不断加强作风建设。牢固树立执政为民思想，把实现好、维护好、发展好人民群众的根本利益作为政府工作的出发点和落脚点，大力弘扬求真务实精神，努力为民多办实事，当好人民公仆。全面落实党风廉政建设责任制，深入推进反腐倡廉工作，大力提高政府工作人员的廉洁自律意识，做到自警、自重、自爱；发扬奉献、清廉、节俭精神，把人民政府建设成为人民满意、人民信任的政府。

各位代表，呼伦贝尔经济社会发展正处在加快转型提升的关键时期，实现呼伦贝尔经济社会又快又好发展，是我们肩负的重大历史责任。让我们在自治区党委、政府和市委的正确领导下，紧紧依靠全市各族人民的智慧和力量，牢牢把握发展机遇，开拓进取，艰苦奋斗，扎实工作，为实现全市国民经济和社会发展第十一个五年规划和全面建设小康社会的宏伟目标而努力奋斗！
